# CCN family member 2/connective tissue growth factor (CCN2/CTGF) is regulated by Wnt–β-catenin signaling in nucleus pulposus cells

**DOI:** 10.1186/s13075-018-1723-8

**Published:** 2018-09-29

**Authors:** Akihiko Hiyama, Kosuke Morita, Daisuke Sakai, Masahiko Watanabe

**Affiliations:** 1 Department of Orthopaedic Surgery, Surgical Science, Tokai University School of Medicine, 143 Shimokasuya, Isehara, Kanagawa 259-1193 Japan; 2 Research Center for Regenerative Medicine, Tokai University School of Medicine, 143 Shimokasuya, Isehara, Kanagawa 259-1193 Japan

**Keywords:** Intervertebral disc, Nucleus pulposus cell, Wnt/β-catenin signaling, CCN2/CTGF, MAPK pathway, Intervertebral disc degeneration

## Abstract

**Background:**

The aims of this study were to investigate the gene expression of CCN family members in rat intervertebral disc (IVD) cells and to examine whether Wnt–β-catenin signaling regulates the expression of CCN family 2 (CCN2)/connective tissue growth factor (CTGF) in rat nucleus pulposus (NP) cells.

**Methods:**

The gene expression of CCN family members were assessed in rat IVD cells using real-time reverse transcription polymerase chain reaction (RT-PCR). The expression pattern of CCN2 was also assessed in rat IVD cells using western blot and immunohistochemical analyses. Gain-of-function and loss-of-function experiments were performed to identify the mechanisms by which Wnt–β-catenin signaling influences the activity of the CCN2 promoter. To further determine if the mitogen-activated protein kinase (MAPK) pathway is required for the Wnt–β-catenin signaling-induced regulation of CCN2 expression in the NP cells, CCN2 expression was analyzed by reporter assay, RT-PCR and western blot analysis.

**Results:**

*CCN2* messenger RNA (mRNA) and protein were expressed in rat IVDs. Expression of *CCN2* was significantly higher than for mRNA of other CCN family members in both rat NP and annulus fibrosus (AF) cells. The relative activity of the CCN2 promoter decreased 24 h after treatment with 6-bromoindirubin-3′-oxime (1.0 μM) (0.773 (95% 0.735, 0.812) *P* = 0.0077) in NP cells. In addition, treatment with the WT–β-catenin vector (500 ng) significantly decreased CCN2 promoter activity (0.688 (95% 0.535, 0.842) *P* = 0.0063), whereas β-catenin small interfering RNA (500 ng) significantly increased CCN2 promoter activity (1.775 (95% 1.435, 2.115) *P* < 0.001). Activation of Wnt–β-catenin signaling decreased the expression of *CCN2* mRNA and protein by NP cells. Regulation of CCN2 by Wnt–β-catenin signaling involved the MAPK pathway in rat NP cells.

**Conclusions:**

This study shows that Wnt–β-catenin signaling regulates the expression of CCN2 through the MAPK pathway in NP cells. Understanding the balance between Wnt–β-catenin signaling and CCN2 is necessary for developing therapeutic alternatives for the treatment of IVD degeneration.

**Electronic supplementary material:**

The online version of this article (10.1186/s13075-018-1723-8) contains supplementary material, which is available to authorized users.

## Background

Low back pain (LBP) is often attributed to intervertebral disc (IVD) degeneration, which is also termed degenerative disc disease. The IVD is composed of a soft nucleus pulposus (NP) surrounded by a tough annulus fibrosis (AF). IVD degeneration occurs during aging and is a complex process, and the underlying mechanical and molecular mechanisms remain poorly understood.

CCN family 2 (CCN2)/connective tissue growth factor (CTGF) is a member of the CCN family of secreted multifunctional proteins, which also includes Cyr61/CCN1, NOV/CCN3, WISP1/CCN4, WISP2/CCN5, and WISP3/CCN6 [1–4]. Of the CCN family members, CCN1 appears to possess activity and an expression pattern similar to that of CCN2, whereas CCN3 appears to act antagonistically to CCN2 [5]. The multimodular character of CCN factors allows multiple interactions between them and other growth factors, such as transforming growth factor β (TGF-β), bone morphogenetic protein (BMP), and insulin-like growth factor, and allows networking between growth factors, the extracellular matrix (ECM), and cell surface receptors such as integrins [6].

CCN2 has emerged as a major regulator of chondrogenesis [7]. Previous studies have shown that the addition of exogenous CCN2 to cultured chondrocytes promotes cell proliferation and the synthesis of proteoglycans concomitant with elevated expression of chondrocyte-associated genes [8, 9]. Other in vivo studies have shown that *Ccn2*-deficient mice die soon after birth as a result of severe skeletal abnormalities associated with impaired chondrocyte proliferation and ECM production [10, 11]. These in vivo findings support the in vitro finding that CCN2 is a promoter of cell proliferation and differentiation during endochondral ossification.

CCN2 protein has also been reported to act within the context of the IVD in vivo and in vitro. For example, CCN2 plays an anabolic role by stimulating matrix production by NP cells [12]. It may be possible to exploit this protein’s activity by including it in a regenerative cocktail delivered to IVDs [12–14]. Interestingly, two groups have reported increased levels of CCN2 protein in degenerated and painful human discs [13, 15]. These reports suggested that CCN2 has some influence on the IVD degeneration process.

There is evidence that the expression of some members of the CCN family, namely CCN2 and CCN4, are regulated by the Wnt–β-catenin signaling [16, 17]. However, the relationship between Wnt–β-catenin signaling and CCN2 in the pathogenesis of IVD disease remains unclear. The aims of this study were (1) to investigate the expression of CCN family members messenger RNA (mRNA) in rat IVD cells and (2) to examine whether Wnt–β-catenin signaling regulate the expression of CCN2 in rat NP cells.

## Methods

### Ethics statement

Animal experiments were performed according to a protocol approved by the Animal Experimentation Committee of the University of Tokai (permit number 131012 and 142055), Tokyo, Japan.

### Reagents and plasmids

To determine the β-catenin–T cell factor (TCF)/lymphoid enhancing factor (LEF) transcription activity after treatment with 6-bromoindirubin-3′-oxime (BIO) (number 361550; Calbiochem, San Diego, CA, USA), NP cells were transiently transfected with the TCF/LEF reporter gene Topflash (optimal TCF binding site) (Upstate Biotechnology). CCN2-luc was provided by Dr Xiaolong Yang (Cornell University, Ithaca, NY, USA). The wild-type (WT) β-catenin expression plasmid and the backbone plasmid (pBI-β-catenin) were provided by Dr. Raymond Poon (Hospital for Sick Children, University of Toronto, Toronto, ON, Canada). The β-catenin small interfering RNA (siRNA) (number sc-29209) and control siRNA duplexes were purchased from Santa Cruz Biotechnology (Santa Cruz, California, CA, USA). WT-pcDNA3-T7–extracellular signal-regulated protein kinase (ERK) 1 (#14440) and WT-pcDNA3-HA–ERK2 (#8974) were purchased from the Addgene repository (Cambridge, MA, USA). FLAG-tagged WT-p38α (WT-p38) was provided by Dr Jiahuai Han (Xiamen University, China).

We used the pGL4.74 vector (Promega, WI, USA) containing the *Renilla reniformis* luciferase gene as an internal transfection control. We used BIO to examine Wnt signaling activity. BIO is a cell-permeable, highly potent, selective, reversible, and ATP-competitive specific inhibitor of glycogen synthase kinase 3α/β activity [18]. The ERK inhibitor (PD98059, #9900) and p38–mitogen-activated protein kinase (MAPK) inhibitor (SB202190, #8158) were obtained from Cell Signaling Technology (Danvers, MA, USA).

### Cell isolation and culture

Rat IVD cells were isolated from multiple levels of lumbar discs of 11-week-old Sprague Dawley rats (*n* = 32). Several samples from the same biological from each of the rats are taken and used as a mass for culturing. Primary rat NP and AF cells were isolated as described [19], and the NP and AF tissues obtained from the same animal were pooled. Isolated cells were maintained in Dulbecco’s modified Eagle medium (DMEM) (Invitrogen, Carlsbad, CA, USA) supplemented with 10% fetal bovine serum (FBS) (Invitrogen) and antibiotics at 37 °C in a humidified atmosphere of 5% CO_2_. Confluent NP and AF cells were harvested and sub-cultured in 10-cm dishes. Low-passage (< 4) cells cultured in monolayers were used for all experiments because cells obtained from the rat IVD tissues exhibited variable morphology until passage 4.

### Human NP tissue specimens

We obtained informed consent from patients for the use of their IVD tissues. The participants’ written consent was obtained according to the Declaration of Helsinki. Ethical approval was obtained from the Institutional Ethics Review Board of the Tokai University School of Medicine. Human degenerative disc tissues were obtained from seven patients undergoing discectomy or fusion surgery at our hospital. We collected seven disc-samples from seven patients (male/female 4/3). The average age of the patients was 38.1 (16–66) years. The details of the samples are listed in Table 1. The disease state was assessed using Pfirrmann grading [20]. This grading scheme uses T2-weighted magnetic resonance imaging (MRI) and image analysis by three independent observers.

**Table 1:** Information on human disc samples from seven patients

Population of seven patients whose samples were used					
Number	Age	Sex	Diagnosis	IVD level	Grade
1	16	F	Disc herniation	L5/6	4
2	18	F	Disc herniation	L4/5	4
3	26	M	Disc herniation	L4/5	5
4	41	M	Disc herniation	L5/S1	4
5	44	M	Disc herniation	L4/5	4
6	56	F	Disc herniation	L3/4	4
7	66	M	Disc herniation	L1/2	5

*M* male, *F* female

Immediately after surgery, human disc NP tissues were carefully collected from discarded surgical waste and digested in 1% penicillin/streptomycin-supplemented DMEM with 10% FBS and 0.114% collagenase type 2 for 1 h at 37 °C. Isolated cells were grown to ~ 80% confluence as a monolayer in 1% penicillin/streptomycin-supplemented DMEM with 10% FBS at 37 °C in a humidified atmosphere of 5% CO_2_. Human NP cells were then used for real-time PCR analysis to evaluate the gene expression of the CCN family members.

### Immunofluorescence staining

Rat NP cells were plated in 96-well flat-bottom plates (3 × 10^3^ cells/well) and incubated for 24 h. The cells were treated with 1.0 μM BIO, fixed with 4% paraformaldehyde, permeabilized with 0.5% Triton X-100 (vol:vol) in phosphate-buffered saline (PBS) for 10 min, blocked with PBS containing 10% FBS, and incubated overnight at 4 °C with antibodies against CCN2 (1:100, Santa Cruz Biotechnology). The cells were washed and incubated with an anti-rabbit Alexa Fluor 488 (green) antibody (Thermo Scientific, IN, USA) at 1:200 and with 10 μM 4′,6-diamidino-2-phenylindole (DAPI) for 1 h at room temperature for nuclear staining. The samples were observed under a fluorescence microscope interfaced with a digital imaging system. Cells treated with normal IgG (Cell Signaling Technology) at equal protein concentrations were used as negative controls.

### Immunohistological studies

To gain insight into the expression of CCN2 in the IVD, freshly isolated spines from 11-week-old (mature) (*n* = 4) and 32-week-old (adult) (n = 4) rats were fixed in 4% paraformaldehyde in PBS, decalcified, and embedded in paraffin wax. The IVD tissue specimen was cut into thin sections that can be placed on unstained slides. At least three different IVD tissue specimens were used from the same individual rat. At least 12 different IVD sections per group were immunohistochemically analyzed. Sagittal sections were deparaffinized in xylene, rehydrated through a graded ethanol series, and stained with hematoxylin. Sections were incubated with antibodies to CCN2 (Santa Cruz Biotechnology) in 2% bovine serum albumin (BSA) in PBS at 1:100 overnight at 4 °C. The sections were washed thoroughly and incubated with a biotinylated universal secondary antibody (Vector Laboratories, Burlington, ON, Canada) at 1:20 for 10 min at room temperature. Sections were incubated with a streptavidin–peroxidase complex for 5 min and washed with PBS, and color was developed using 3,3′-diaminobenzidine (Vectastain Universal Quick Kit; Vector Laboratories) and examined under a fluorescence microscope. Non-immune IgG was used as a negative control (Cell Signaling Technology) and mouse ovary was used as positive control. The number of positively immunolabeled cells and the total number of cells per high-power field in each section were determined, and the percentage of positively labeled cells was calculated.

### Real-time reverse RT–PCR analysis

Total RNA was extracted from the cells using the TRIzol RNA isolation protocol (Invitrogen). RNA was treated with RNase-free DNAse I. Total RNA (100 ng) was used as the template for the RT-PCR analyses. Complementary DNA (cDNA) was synthesized via the reverse transcription of mRNA, as described previously [21]. Reactions were arranged in triplicate in 96-well plates using 1 μL of cDNA with SYBR Green PCR Master Mix (Applied Biosystems), to which gene-specific forward and reverse PCR primers for BIO were added. The primers were synthesized by Takara Bio Inc. (Tokyo, Japan) or FASMAC Corp. (Tokyo, Japan) and are shown in Table 2. PCR reactions were performed in an Applied Biosystems 7500 Fast system according to the manufacturer’s instructions. The expression scores were obtained using the ΔΔC_t_ calculation method. The relative quantification of gene expression in the treatment groups versus control (cells isolated freshly before culture) was performed using the comparative threshold cycle method:

**Table 2:** Primers for real-time PCR

Target	NCBI number	Forward primer, 5′- 3’	Reverse primer, 5′- 3’
CCN1 (CYR61)	NM_031327.2	TCACTGAAGAGGCTTCCTGTC	CCAGTTCCGCAGCTCTTG
CCN2 (CTGF)	NM_022266.2	GCTGACCTAGAGGAAAACATTAAGA	CCGGTAGGTCTTCACACTGG
CCN3 (NOV)	NM_030868.2	CGGCCTTGTGAGCAAGAG	TTCTTGGTCCGGAGACACTT
CCN4 (Wisp1)	NM_031716.1	ACATCCGACCACACATCAAG	AAGTTCGTGGCCTCCTCTG
CCN5 (Wisp2)	NM_031590.1	CAGGGCCTGGTTTGTCAG	CCGTCATCCTCATCCAAGA
CCN6 (Wisp3)	NM_001170483.1	CATGGAAGGCAGGGAAGA	CTTTGGGGAGTTGGAAAGTG

2^[(C_tGAPDH_–C_tGene_)_treatment_–(C_tGAPDH_– C_tGene_)_control_],

where glyceraldehyde 3-phosphate dehydrogenase (GAPDH) was used as the housekeeping control gene. GAPDH is has good feasibility as an endogeneous control for IVD cells [22].

### Western blot analysis

Treated rat NP cells were immediately placed on ice and washed with cold PBS. To prepare the total cellular proteins, the cells were lysed with lysis buffer containing 10 mM Tris-HCl (pH 7.6), 50 mM NaCl, 5 mM EDTA, 1% Nonidet P-40, complete protease inhibitor cocktail (Roche, IN, USA), 1 mM NaF, and 1 mM Na_3_VO_4_. Heat-denatured samples were separated on sodium dodecyl sulfate polyacrylamide gels and electrotransferred onto Immobilon-P polyvinylidene difluoride membranes (Millipore, MA, USA). The membranes were then blocked with blocking buffer (5% BSA and 0.1% NaN_3_ in PBS) and subsequently incubated overnight at 4 °C with anti-CCN2 (1:1000, Santa Cruz Biotechnology or Abcam, Cambridge, UK) antibodies diluted in Can Get Signal Immunoreaction Enhancer Solution (Toyobo, Tokyo, Japan). Chemiluminescent signals were visualized with an Immobilon Western Chemiluminescent HRP Substrate (Millipore) and scanned using an Ez-Capture MG imaging system (ATTO, Tokyo, Japan). The western blot data were quantified using Image J pixel analysis (NIH Image software). Western blot data are presented as band intensity normalized to that of the loading control (β-actin). To measure the band intensity, the data shown are representative of at least three independent experiments.

### Gene-suppression studies using siRNA

We silenced β-catenin expression in NP cells using siRNA technology. In brief, NP cells were transferred into 24-well plates at a density of 6 × 10^4^ cells/well 1 day before transfection. The next day, cells were treated with β-catenin siRNA or control siRNA duplexes at a final concentration of 100–500 ng/ml using Lipofectamine 2000 (Invitrogen). Cells also received CCN2 promoter constructs and the pGL4.74 plasmid at the time of transfection. At 6 h after transfection, the medium was replaced with complete growth medium, and the cells were allowed to recover for 18 h. Cells were then cultured for 24 h and luciferase activity was measured.

### Transfections and Dual-Luciferase™ assay

Rat NP and AF cells were transferred to 24-well plates at 3 × 10^4^ cells/well 1 day before transfection. Cells were cotransfected with 100–500 ng of expression plasmids or the backbone vector together with the reporter plasmids. Lipofectamine 2000 (Invitrogen) was used as the transfection reagent. Reporter activity was measured 48 h after transfection using the Dual-Luciferase™ reporter assay system (Promega) for the sequential measurements of *Firefly* and *Renilla* luciferase activities. The results were normalized to the transfection efficiency and are expressed as the ratio of luciferase to pGL4.74 activity (denoted as “relative activity”). NP and AF cells were transfected with a plasmid encoding green fluorescent protein to check the transfection efficiency, which was 60–70% in NP cells. Luciferase activity and relative ratio were quantified using a Turner Designs Luminometer Model TD-20/20 instrument (Promega).

### Statistical analysis

All experiments were performed at least three times or more, and the experiment was replicated at least twice each time. The data are expressed as the mean ± 95% confidence interval (CI). Student’s *t* test and the Mann-Whitney U test were used to compare two groups, and one-way analysis of variance (ANOVA) to compare three or more groups. The positivity of protein and gene expression was analyzed by unpaired Student’s *t* test. One-way ANOVA was used to identify significant differences in transcription levels between the study groups. Statistical analyses were performed using SPSS software (ver. 12.0; SPSS Corp., IL, USA). Significance was set at *P* < 0.05.

## Results

### Expression of CCN family members in IVD cells

**Fig. 1:**
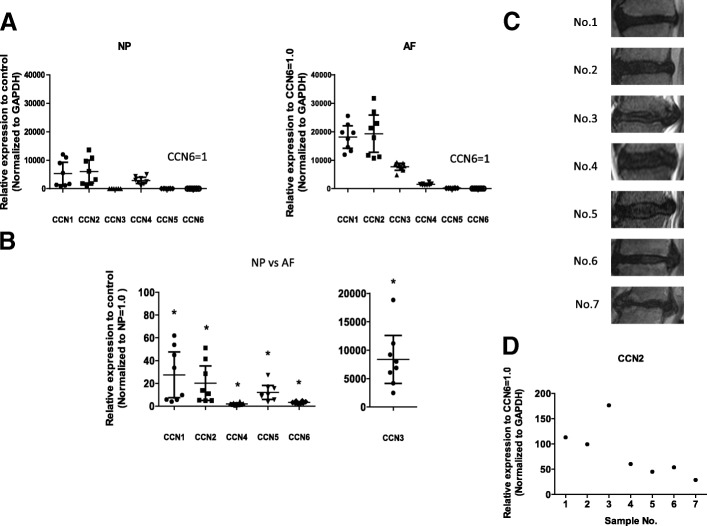
Expression of CCN family members in rat intravertebral disc (IVD) cells. **a** Real-time RT-PCR analysis of mRNA expression of CCN family members in rat nucleus pulposus (NP) and annulus fibrosus (AF) cells. Results are presented as mean and 95% CI as the fold change relative to the CCN6 (= 1.0) (*n* = 8 for each group) both in NP versus AF cells. **b** Comparison of mRNA expression of CCN family members in NP and AF cells assessed using real-time RT-PCR analysis. Data are the mean and 95% CI (*n* = 8). The unpaired Student’s *t* test was used. **c** Lumbar magnetic resonance imaging findings in seven patients. The IVD was low-intensity on T2-weighted images. The IVD was grade 4 in five patients grade 5 in two patients. **d** Real-time RT-PCR analysis of mRNA expression of CCN2 in human NP cells. Glyceraldehyde 3-phosphate dehydrogenase (GAPDH) was used as an endogenous control

**Fig. 2:**
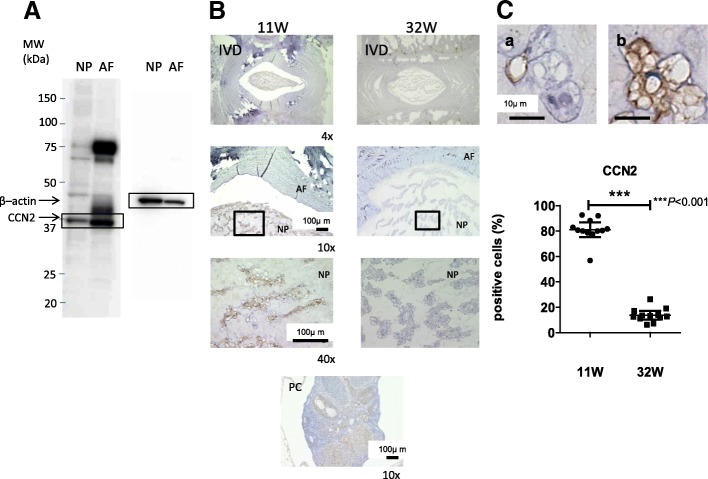
Expression of CCN2 in rat intravertebral discs (IVDs). **a** Western blot analysis of CCN2 protein in rat nucleus pulposus (NP) and annulus fibrosus (AF) cells. β-actin was used as a loading control. Immunoblots shown are representative of experiments with similar results (*n* = 8). The unpaired Student’s *t* test was used. **b** Immunohistological staining of CCN2 expression in sagittal sections from 11-week-old (mature) and 32-week-old (adult) rats. Rat kidney was used as positive control (PC). Scale bar = 20–500 μm (× 4–40 original magnification). **c** CCN2 negative (a) and positive (b) cells were immunohistochemically detected in rat NP cells. Cells stained brown are positive cells. The values are the number of CCN2-positive NP cells. The percentage of cells positive for CCN2 in NP cells was calculated from the staining (*n* = 12). The unpaired Student’s *t* test was used. Scale bar = 10 μm

We evaluated the expression of all CCN members in cultured rat IVD cells at the mRNA level using real-time PCR (Fig. 1a). The mRNA expression of all CCN family members was significantly higher in rat AF than in rat NP cells. In particular, expression of *CCN3* mRNA was more prominent in AF cells than NP cells (9560-fold, *P* < 0.001). In addition, the expression level was significantly higher for *CCN2* mRNA than for mRNA of other CCN family members in both rat NP and rat AF cells (Fig. 1b). Next, we measured the gene expression levels of the CCN family members in the human NP samples. The representative MRI images of discs of different age and different levels of degeneration are shown (Fig. 1c). The gene expression of CCN family members was also confirmed, but the gene expression levels of CCN family members except *CCN1* mRNA and *CCN2* mRNA were equivalent in human NP cells (data not shown). Real-time PCR showed a higher level of the *CCN2* mRNA compared with other CCN family members mRNA in the human NP samples. It is unclear because there were few samples, but it was suggested that expression of *CCN2* mRNA may be lower as age increases (Fig. 1d).We also evaluated the expression of CCN2 protein in cultured rat IVD cells using western blot analysis. Figure 2A shows that both NP and AF cells expressed a prominent 38 kDa CCN2 band. The expression level of CCN2 protein was higher in AF than in NP cells. The expression of CCN2 protein in IVDs from 11 weeks and 32 weeks rats was immunohistochemically examined. CCN2 was expressed in NP and AF cells in discs from 11-week-old rats. However, there was weak expression of CCN2 protein in IVDs from the 32-week-old rats (Fig. 2B). That is, the percentage of cells in the rat NP that were immunopositive for CCN2 decreased significantly with age (11 weeks, 80.9 ± 9.1%; 32 weeks, 13.7 ± 5.3%; *P* < 0.001).

### CCN2/CTGF is regulated by Wnt–β-catenin signaling

To determine whether CCN2 expression is modulated by Wnt–β-catenin signaling in rat NP cells, NP and AF cells were cultured with BIO (0–1.0 μM). We first examined activation of Wnt by BIO stimulation. The results showed that there was an increase in the activity of Topflash upon BIO stimulation (Additional file 1: Figure S1).

**Fig. 3:**
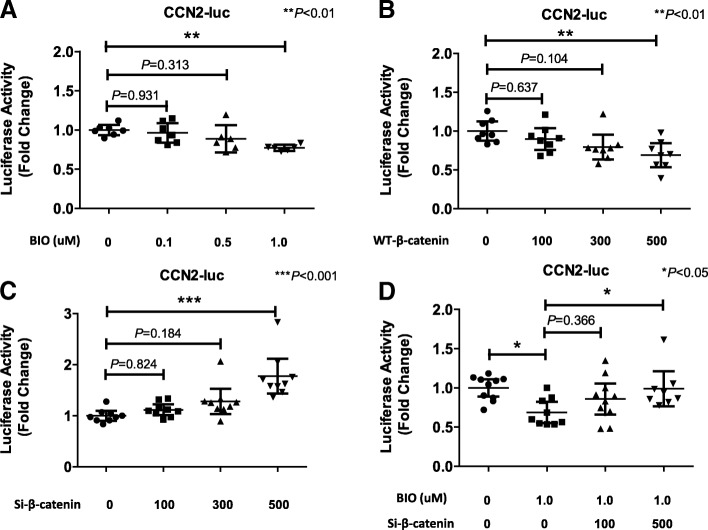
Effect of Wnt–β-catenin signaling on CCN2 expression in rat nucleus pulposus (NP) cells. **a** Rat NP cells transfected with the CCN2 reporter plasmid together with the pGL4.74 plasmid were treated with different concentrations (0, 0.1, 0.5, 1.0 μM) of 6-bromoindirubin-3′-oxime (BIO) for 24 h. **b**, **c** NP cells were cotransfected with the CCN2 reporter plasmid together with WT-β-catenin (**b**), β-catenin siRNA (si-β-catenin) (**c**), or empty vectors and the pGL4.74 vector. Cells were cultured for 24 h and luciferase (luc) reporter activity was measured. The results were normalized for transfection efficiency and are expressed as the ratio of luciferase relative to pGL4.74 activities (denoted as relative activity). **d** NP cells transfected with the CCN2 reporter plasmid were treated with the β-catenin siRNA (si-β-catenin), which was added during exposure of the cells to 1.0 μM BIO. Results are presented as mean and 95% CI (*n* = ≥ 8 for each group). One-way analysis of variance with the Tukey-Kramer post-hoc test was used for all experiments

The activity of the CCN2 promoter decreased 24 h after BIO treatment (1.0 μM) in both NP cells (*P* = 0.0077) (Fig. 3a) and AF cells (*P* = 0.0236) (Additional file 2: Figure S2). In another experimental approach, we transfected rat NP cells with the WT-β-catenin vector or β-catenin siRNA 24 h before the experiments. Treatment with the WT-β-catenin vector significantly decreased CCN2 promoter activity (500 ng) (Fig. 3b) (*P* = 0.0063), whereas β-catenin siRNA significantly increased CCN2 promoter activity (500 ng) (*P* < 0.001) (Fig. 3c). Next, rat NP cells were pretreated with β-catenin siRNA together with a control vector or BIO (0–1.0 μM) 24 h before the experiments. Treatment with β-catenin siRNA significantly attenuated the BIO-induced decrease in CCN2 promoter activity (*P* = 0.0419) (Fig. 3d). These results suggest that Wnt–β-catenin signaling regulates the expression of CCN2 at the transcriptional level.

**Fig. 4:**
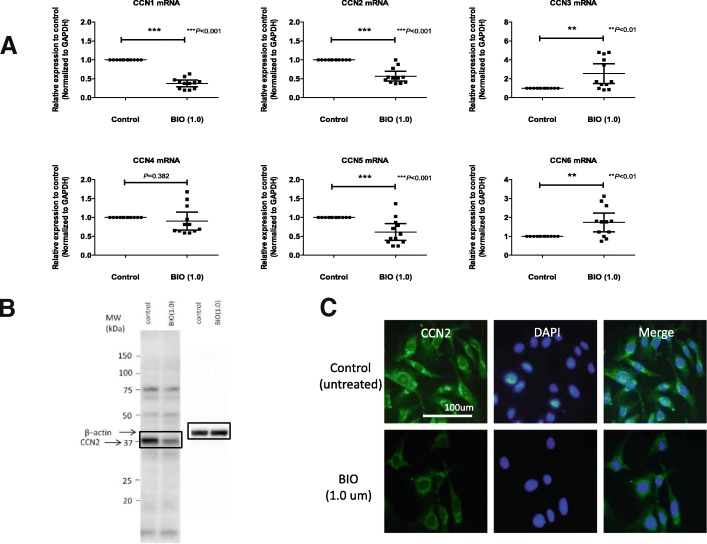
Effect of Wnt–β-catenin signaling on CCN2 mRNA and protein expression in rat nucleus pulposus (NP) cells. **a** mRNA expression of CCN members (CCN1, CCN2, CCN3, CCN4, CCN5, and CCN6) after exposure of NP cells to 6-bromoindirubin-3′-oxime (BIO) (1.0 μM) for 24 h assessed using real-time PCR. Results are presented as mean and 95% CI (*n* = 12 for each group). Glyceraldehyde 3-phosphate dehydrogenase (GAPDH) was used as an endogenous control. The unpaired Student’s *t* test was used. **b** Western blot analysis of CCN2 protein after treatment of NP cells with BIO (1.0 μM). Western blot analysis showed there was a decrease in the levels of CCN2 protein after BIO treatment. β-actin was used as a loading control. Immunoblots shown are representative of experiments with similar results (*n* = 8). The paired Student’s *t* test was used. **c** Detection of CCN2 protein expression by immunofluorescence microscopy. NP cells were cultured with or without 1.0 μM BIO for 24 h, fixed, and stained with antibody against CCN2. CCN2 protein is decreased with treatment compared with untreated control. Left: cells stained with antibody to CCN2; middle: cells stained with 4′,6-diamidino-2-phenylindole (DAPI) to identify healthy nuclei; right: cells stained with antibody to CCN2 and DAPI. Scale bar = 100 μm (× 20 original magnification)

To confirm the reporter assay data, real-time PCR analysis was performed to analyze the gene expression of CCN family members in both NP and AF cells. As expected, BIO treatment significantly decreased the expression of *CCN1*, *CCN2*, and *CCN5* mRNA at 24 h in NP cells (all *P* < 0.001), while *CCN3* (*P* = 0.0037, *P* < 0.01) and *CCN6* (*P* = 0.0037, *P* < 0.01) mRNA were increased after the BIO treatment (Fig. 4a). Similar results of *CCN1*, *CCN2* and *CCN5* mRNA were obtained with AF cells (Additional file 3: Figure S3). Western blot analysis of cell lysates also indicated that CCN2 protein levels were decreased at 24 h after BIO treatment (Fig. 4b). The expression of the CCN2 protein was further assessed using immunofluorescence microscopy in the BIO-treated NP cells at 24 h after BIO treatment. As shown in (Fig. 4c), CCN2 protein expression was decreased after BIO treatment compared with the untreated control.

### Suppression of CCN2 by Wnt–β-catenin signaling through the MAPK pathways in NP cells

**Fig. 5:**
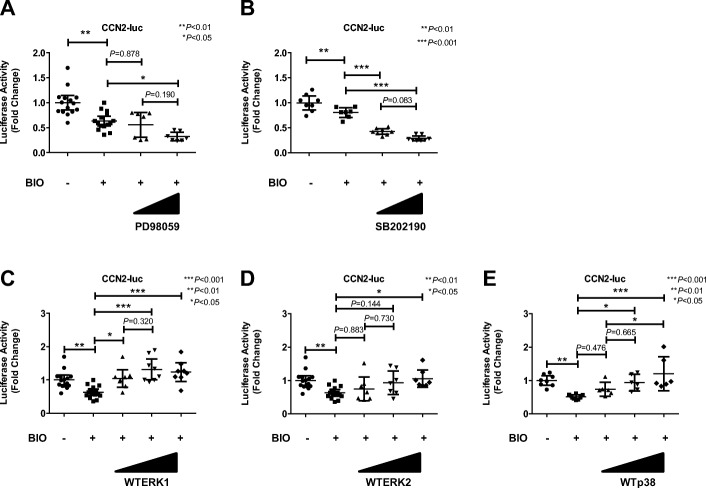
Wnt–β-catenin signaling suppress CCN2 expression through the mitogen-activated protein kinase (MAPK) pathway in rat nucleus pulposus (NP) cells. **a**-**b** NP cells transfected with the CCN2 reporter plasmid together with the pGL4.74 plasmid were treated with 6-bromoindirubin-3′-oxime (BIO) (1.0 μM) and the extracellular signal-related protein kinase (ERK) inhibitor (PD98059, 25 or 50 μM) (**a**) or the p38–MAPK inhibitor (SB202190, 1 or 10 μM) (**b**). **c**-**e** Rat NP cells transfected with the CCN2 reporter plasmid were cotransfected with different concentrations of the wild-type (WT)-ERK1 (**c**), (**d**) WT-ERK2, or (**e**) WT-p38 expression vector together with BIO (1.0 μM). Results are presented as mean and 95% CI (*n* = ≥ 6 for each group). One-way analysis of variance with the Tukey-Kramer post-hoc test was used for all experiments

**Fig. 6:**
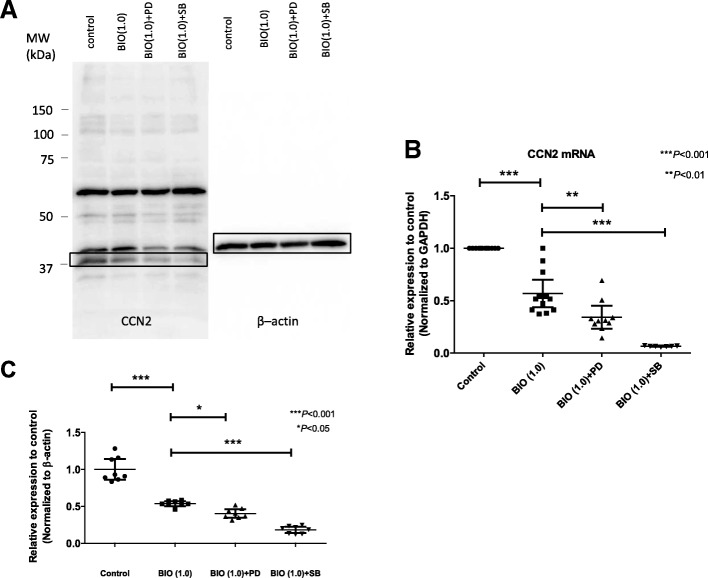
Regulation of the CCN2 gene and protein by Wnt–β-catenin signaling via the mitogen-activated protein kinase (MAPK) pathway. **a** Western blot analysis of the CCN2 protein in nucleus pulposus (NP) cells after treatment with 6-bromoindirubin-3′-oxime (BIO) with the extracellular signal-related protein kinase (ERK) inhibitor PD98059 (PD) (25 μM) or the p38–MAPK inhibitor SB202190 (SB) (10 μM) in rat NP cells. β-actin was used as a loading control. Data are the mean and 95% CI. One-way analysis of variance with the Tukey-Kramer post-hoc test was used. Immunoblots shown are representative of experiments with similar results (*n* = 8 for each group). **b** Real-time RT-PCR analysis of CCN2 mRNA expression after treatment with BIO with the ERK inhibitor PD98059 (25 μM) or the p38–MAPK inhibitor SB202190 (10 μM) in rat NP cells. Data are the mean and 95% CI expressed as the fold change relative to the control (*n* = ≥ 7 for each group). **c** Densitometric analysis as shown (A) confirms that BIO treatment significantly decreased the activity of the CCN2 protein and this activity was suppressed by the MAPK inhibitors treatment. One-way ANOVA with the Tukey-Kramer post-hoc test was used

To further determine if the MAPK pathway is required for the Wnt–β-catenin signaling-induced regulation of CCN2 expression in the NP cells, we evaluated the activation of the Wnt–β-catenin signaling following treatment with specific inhibitors of ERK1/2 (PD98059) or p38–MAPK (SB202190). NP cells transfected with the CCN2 reporter plasmid together with the pGL4.74 plasmid were treated with specific inhibitors of the MAPK pathway after the BIO (1.0 μM) treatment for 24 h. Figure 5 (a, b) shows that BIO treatment significantly decreased the activity of the CCN2 promoter; this activity was suppressed by the PD98059 (20 μM, *P* = 0.017) and SB202190 (1 μM and 10 μM, *P* < 0.001). To investigate the role of the MAPK pathway in Wnt–β-catenin signaling downregulation of CCN2, we further transfected rat NP cells with WT vector (ERK1, ERK2, and p38) together with BIO, and measured CCN2 promoter activity. The promoter activity of CCN2 was suppressed by BIO treatment, but it was reverse-activated by transfection of WT-ERK1 (Fig. 5c), WT-ERK2 (Fig. 5d) and WT- p38 (Fig. 5e). These results showed that ERK1, ERK2, and p38 abolished the suppression of the transcriptional activity of CCN2 in NP cells treated with BIO. Real-time PCR and western blot analysis also showed that BIO treatment combined with MAPK inhibitors (PD98059 or SB202190) decreased the expression of CCN2 levels further (Fig. 6a) and Fig. 6b).

Densitometric analysis confirms that the MAPK pathway is involved in suppression of CCN2 by Wnt–β-catenin signaling (Fig. 6c). BIO treatment significantly decreased the expression of the *CCN2* mRNA; this activity was not suppressed completely by the JNK inhibitor SP600125 (data not shown). Thus, we did not investigate JNK in this study.

## Discussion

The experiments demonstrated for the first time that CCN2 expression in IVD cells is regulated by Wnt–β-catenin signaling. We found that rat IVD cells expressed CCN family members and activation of Wnt–β-catenin signaling reduced activity of the CCN2 promoter, gene, and protein levels in rat NP cells. We have already reported on the involvement of the MAPK pathway in Wnt–β-catenin signaling [23]. Subsequently, we found that Wnt–β-catenin signaling suppressed CCN2 expression in rat NP cells, and the expression of CCN2 was regulated by Wnt–β-catenin signaling via the MAPK pathway in this study.

CCN2 is a cysteine-rich secretory protein of 36–38 kd containing 349 amino acid residues. It contains a von Willebrand–type C domain that interacts with growth factors such as transforming growth factor (TGF)-β–bone morphogenic proteins (BMPs) and thereby mediates ECM interactions. Erwin et al. found that notochord cells secrete CCN2 and that conditioned medium obtained from these cells upregulates important matrix gene expression, cell proliferation, and proteoglycan production in NP cells [24]. They also suggested that the loss of TGF-β1 and CCN2 is associated with the progression of IVD degeneration [25]. Another group, Oh et al., examined whether CCN2 is regulated by SOX9 in IVD tissues. They not only found that CCN2 expression is regulated directly by the transcription factor SOX9 in chondrocytes, they also detected the SOX9 binding site in NP cells [26]. Although CCN2 is involved in anabolic factors, it is also involved with inflammatory cytokines. Tran et al. reported that interleukin 1β (IL-1β) and tumor necrosis factor α (TNF-α) suppress CCN2 expression through the nuclear factor-κB signaling pathway in NP cells [12]. These anabolic and catabolic effects suggest that CCN2 may be an important factor in the pathogenesis of IVD disease. Furthermore, from studies using notochord-specific CCN2-null mice, Bedore et al. found that loss of CCN2 in notochord-derived cells resulted in impaired development of IVDs and marked acceleration of age-associated IVD degeneration [27].

Although the involvement of CCN2 in IVD cells has been studied, its regulation remains unknown. Previous studies have demonstrated that Wnt–β-catenin signaling plays a major role in IVD metabolism [19, 28, 29]. Generally, elevated levels of proinflammatory cytokines and other inflammatory mediators, including TNF-α, IL-1β, IL-6, and prostaglandin E_2_, are present in degenerating IVDs [30, 31]. We have reported that Wnt–β-catenin signaling regulates TNF-α and that Wnt signaling and TNF-α formed a positive-feedback loop in NP cells [32]. We speculated that blocking the Wnt–β-catenin signaling might protect NP cells against degeneration. However, its relationship with Wnt–β-catenin signaling in the pathogenesis of IVD disease remains unclear. Therefore, the objective of this study was to determine whether CCN2 can be regulated by Wnt–β-catenin signaling.

We first examined the expression and localization of specific CCN family members in rat IVD tissues. The expression of *CCN2* mRNA was significantly higher than that of the mRNA of other CCN family members in both rat NP and AF cells. However, a previous report showed that CCN2 expression is higher in the NP than in the AF and that CCN2 levels are higher in the NP of mature rat discs than in neonatal tissues [13]. This result contrasts with ours because it does not depend on individual differences or age. It may be necessary to perform the same analyses in a larger number of human samples. However, we believe that it is not as important to determine whether CCN2 expression is higher in AF or NP cells as it is to confirm that this expression occurs in both cells.

Furthermore, gain-of-function and loss-of-function experiments were performed to identify the mechanism by which Wnt–β-catenin signaling influences the activity of the CCN2 promoter. The results showed that CCN2 promoter activity was regulated by Wnt–β-catenin signaling in NP cells. We speculate that blocking the Wnt–β-catenin signaling might protect NP cells against degeneration by activating CCN2, which stimulates both the proliferation of cells and synthesis of the ECM. We also investigated whether the MAPK pathway is involved in this process. Gain-of-function and loss-of-function experiments were analyzed in the same way. MAPK overexpression increased CCN2 promoter activity, whereas inhibition of the MAPK pathway inhibited its activity. We obtained similar results in gene and protein analysis.

There are several limitations of the present study. First, regulation of the CCN family by Wnt–β-catenin signaling differs between human and rat IVD cells. Animal species and humans differ in many ways in terms of cell populations, anatomy, development, physiology, and mechanical properties of the spine. The central region of the IVD in infant humans is made and maintained by notochordal cells, which disappear during maturation and are replaced by mature chondrocyte-like cells. The disappearance of notochordal cells precedes the onset of IVD degeneration, but whether the disappearance of these cells might be involved in initiating IVD degeneration remains unclear. However, human material is difficult to obtain because of ethical and government regulatory restrictions. Second, as ours is the first report, we conducted the research with simple small animals for analysis. For these reasons, we used normal rat NP cells in several experiments. Third, although we performed cell culture under normoxia, it is well-known that IVD-typical conditions are hypoxic with specific and important effects on cell metabolism. It has been suggested that NP cells are specifically adapted to a hypoxic environment, suggesting that interactions between chemical microenvironment and hypoxia are important questions deserving further study. However, it is very difficult to conclude from all of these study data that low oxygen would be good for the IVD, because there are complex signaling networks involved in IVD degeneration. Finally, we investigated the expression levels of CCN family members in IVD cells and also compared those in the AF and the NP. This showed that the mRNA expression for all CCN family members especially CCN3 was significantly higher in rat AF than in rat NP cells. Thus, we think that further analysis of other CCN family members is necessary, including CCN3.

## Conclusion

We found that the expression of CCN2 was regulated by Wnt–β-catenin signaling in IVD cells. That is, activation of Wnt–β-catenin signaling suppressed the expression of CCN2, suggesting the possibility that the MAPK pathway may be involved in this process. However, further studies are needed to investigate whether Wnt–β-catenin signaling has potential as a treatment target for IVD degeneration in vivo.

## Additional files

Additional file 1: **Figure S1.** Rat NP cells were cotransfected with the Topflash reporter plasmid and pGL4.74 plasmid treated with 1.0 μM of 6-bromoindirubin-3′-oxime (BIO) for 24 h. (PDF 10 kb)

Additional file 2: **Figure S2.** Rat AF cells transfected with the CCN2 reporter plasmid together with the pGL4.74 plasmid were treated with different concentrations (0, 0.1, 1.0 μM) of 6-bromoindirubin-3′-oxime (BIO) for 24 h. (PDF 10 kb)

Additional file 3: **Figure S3.** mRNA expression of CCN members (CCN1, CCN2, CCN3, CCN4, CCN5, and CCN6) after exposure of AF cells to 6-bromoindirubin-3′-oxime (BIO) (1.0 μM) for 24 h assessed using real-time PCR. Results are presented as mean and 95% CI (*n* = 12 for each group). GAPDH was used as an endogenous control. The unpaired Student’s *t* test was used. (PDF 14 kb)

## Abbreviations

AF: Annulus fibrosus
ANOVA: Analysis of variance
BIO: 6-Bromoindirubin-3′-oxime
BMP: Bone morphogenetic proteins
cDNA: Complementary DNA
CTGF: Connective tissue growth factor
ERK: Extracellular signal-regulated protein kinase
FBS: Fetal bovine serum
GAPDH: Glyceraldehyde 3-phosphate dehydrogenase
IL: Interleukin
IVD: Intervertebral disc
LBP: Low back pain
kDa: KiloDalton
MAPK: Mitogen-activated protein kinase
MRI: Magnetic resonance imaging
mRNA: Messenger RNA
PBS: Phosphate-buffered saline
RT-PCR: Reverse transcription polymerase chain reaction
siRNA: Small interfering RNA
TGF-β: Transforming growth factor β
TNF: Tumor necrosis factor
WT: Wild type
