# Supplementary figures and images for: CCN family member 2/connective tissue growth factor (CCN2/CTGF) is regulated by Wnt–β-catenin signaling in nucleus pulposus cells
Source: Arthritis Res Ther. 2018 Sep 29;20:217. doi: 10.1186/s13075-018-1723-8 (PMC6162946; doi:10.1186/s13075-018-1723-8)

Fig. S1

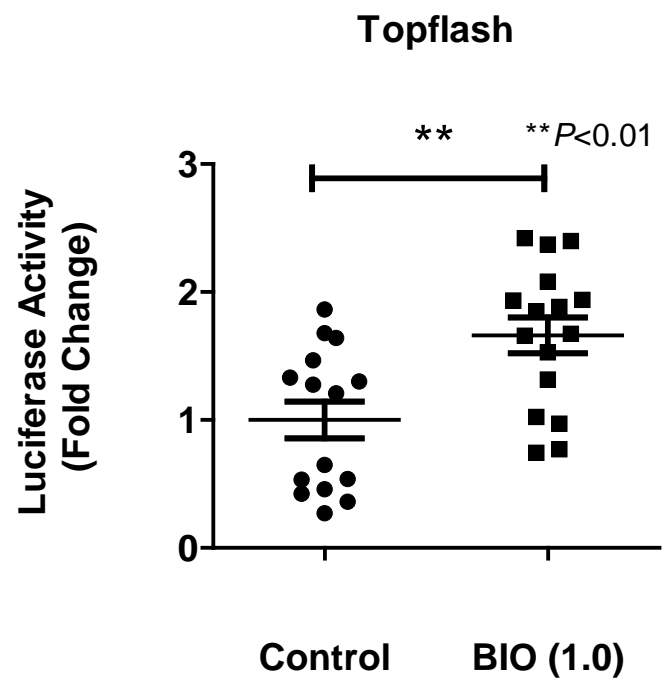

Supplement: Supplementary file 1 — Figure S1. Rat NP cells were cotransfected with the Topflash reporter plasmid and pGL4.74 plasmid treated with 1.0 μM of 6-bromoindirubin-3′-oxime (BIO) for 24 h. (PDF 10 kb) [file 13075_2018_1723_MOESM1_ESM.pdf]

Fig. S2

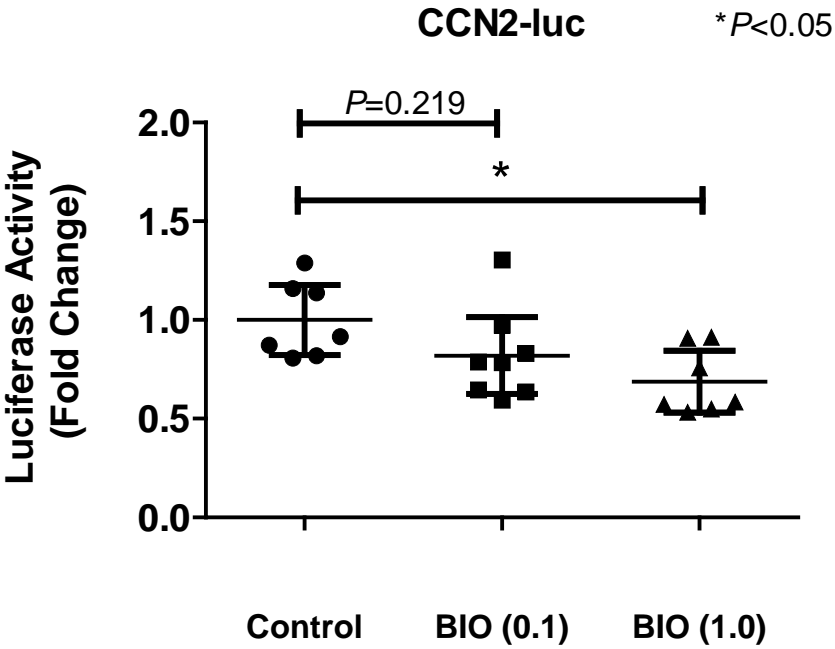

Supplement: Supplementary file 2 — Figure S2. Rat AF cells transfected with the CCN2 reporter plasmid together with the pGL4.74 plasmid were treated with different concentrations (0, 0.1, 1.0 μM) of 6-bromoindirubin-3′-oxime (BIO) for 24 h. (PDF 10 kb) [file 13075_2018_1723_MOESM2_ESM.pdf]

Fig. S3

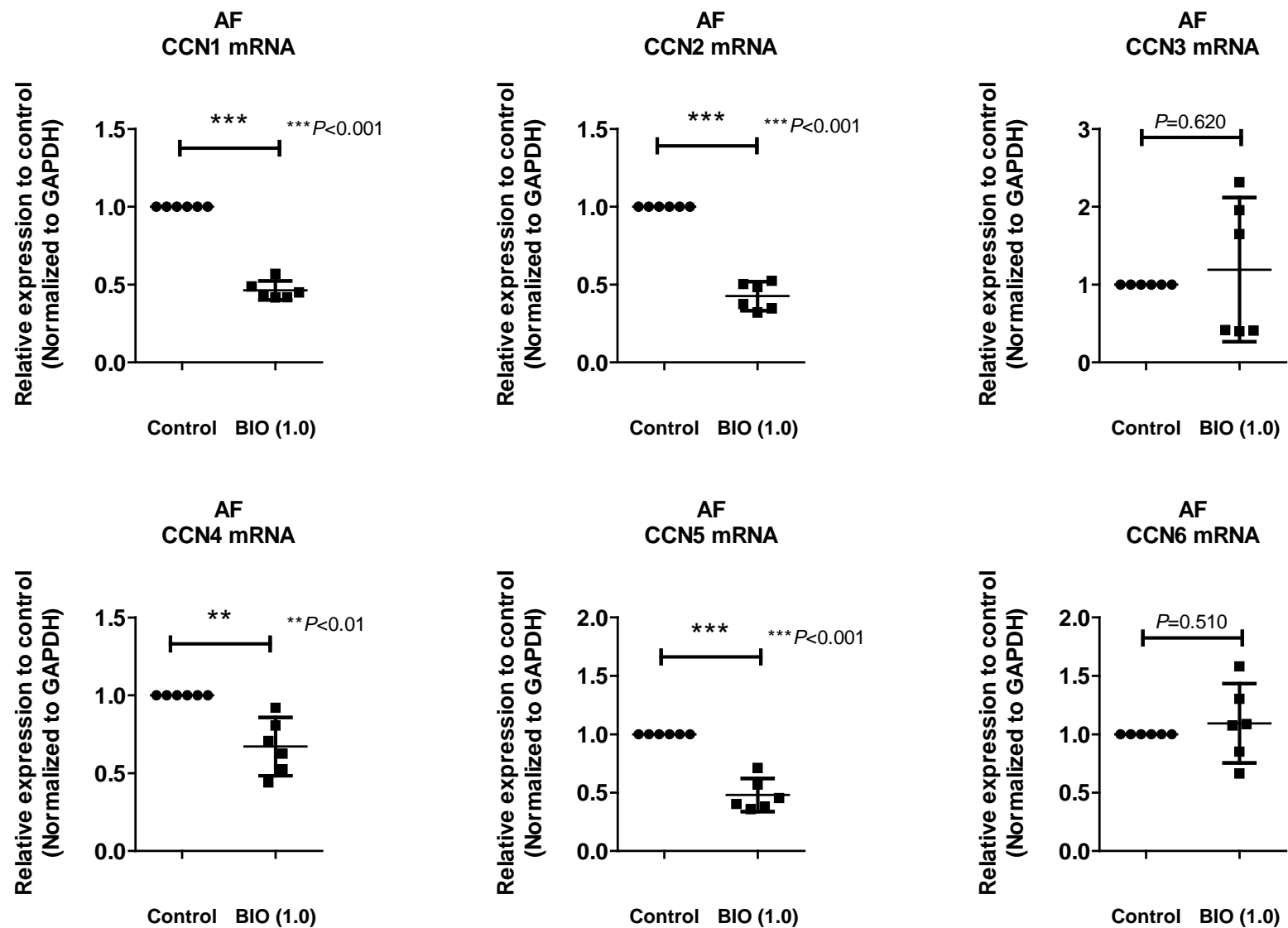

Supplement: Supplementary file 3 — Figure S3. mRNA expression of CCN members (CCN1, CCN2, CCN3, CCN4, CCN5, and CCN6) after exposure of AF cells to 6-bromoindirubin-3′-oxime (BIO) (1.0 μM) for 24 h assessed using real-time PCR. Results are presented as mean and 95% CI (n = 12 for each group). GAPDH was used as an endogenous control. The unpaired Student’s t test was used. (PDF 14 kb) [file 13075_2018_1723_MOESM3_ESM.pdf]
